# Bladder Sparing Extirpation of Pelvic Mass With Ureteral Reimplantation: A Case Report on Pelvic Lipomatosis Treatment

**DOI:** 10.7759/cureus.42047

**Published:** 2023-07-17

**Authors:** Abdulaziz A Albalawi, Abdullatif M Alhassan, Abdullah B Alanazi, Abdullah M Alghamdi

**Affiliations:** 1 Urology, Prince Sultan Military Medical City, Riyadh, SAU; 2 Urology, Security Forces Hospital, Riyadh, SAU

**Keywords:** bilateral hydroureteronephrosis, ureteral reimplantation, mass extirpation, pelvic lipomatosis, bladder sparing surgery

## Abstract

Pelvic lipomatosis is a proliferative disease characterised by excessive fat growth in retroperitoneal space leading to inadequate bladder drainage and ureteral compression. Cystitis glandularis, cystitis cystica, or cystitis follicularis can be found in the majority of patients with the disease.

We report a case of a 63-year-old man diagnosed outside our hospital with pelvic lipomatosis after finding a pelvic mass behind the bladder causing severe bilateral hydronephrosis. A bladder-sparing excision of the pelvic lipomatosis mass with bilateral ureteric reimplantation was performed, thereby avoiding the need for urinary diversion. Our case supports the hypothesis that pelvic fat mass extirpation and ureteral reimplantation is an effective surgical treatment strategy for pelvic lipomatosis.

## Introduction

In the latter half of the 1950s, Engles discovered a rare condition called pelvic lipomatosis now. The disease is proliferative, characterised by excessive fat growth in the retroperitoneal space of the pelvis [1]. Cystitis glandularis, cystitis cystica, or cystitis follicularis can be found in more than 70% of patients diagnosed with pelvic lipomatosis [2].

There is limited research on the disease itself and its long-term effects, and so there are no set guidelines for patients to follow. There are numerous treatment choices, such as watchful waiting or surgery that may involve a total cystectomy or urinary diversion. There have been recent reports of success found in a bladder-sparing technique [3].

We report a case of a 63-year-old man diagnosed outside our hospital with pelvic lipomatosis after finding a pelvic mass behind the bladder causing severe bilateral hydronephrosis. A bladder-sparing excision of the pelvic lipomatosis mass with bilateral ureteric reimplantation was performed, thereby avoiding the need for urinary diversion. Our case supports the hypothesis that pelvic fat mass extirpation and ureteral reimplantation is an effective surgical treatment for pelvic lipomatosis. This article was previously posted to the Research Square preprint server on June 13, 2023.

## Case presentation

A 63-year-old African male presented with a chronic history of lower abdominal pain for more than four years not associated with lower urinary tract symptoms. The patient was diagnosed outside our hospital with pelvic lipomatosis after finding a pelvic mass behind the bladder causing severe bilateral hydronephrosis. He underwent partial pelvic mass excision with bilateral double J stent insertion last year. The patient was referred to our urology department for a second opinion. Histopathology slides were re-submitted and confirmed the diagnosis. His laboratory investigations showed creatinine 109 µmol/L.

**Figure 1:**
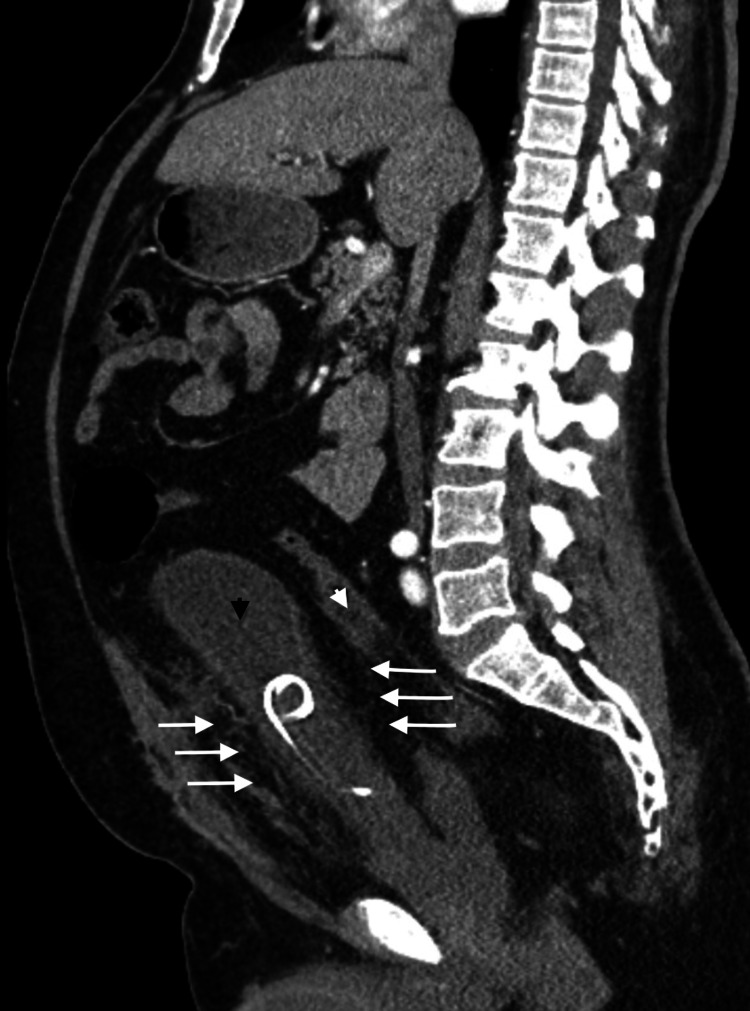
Pre-operative CT (sagittal view) Pear-shaped elongated urinary bladder (black arrowhead) and compressed rectum (white arrowhead) secondary to extensive pelvic lipomatosis (white arrows).

**Figure 2:**
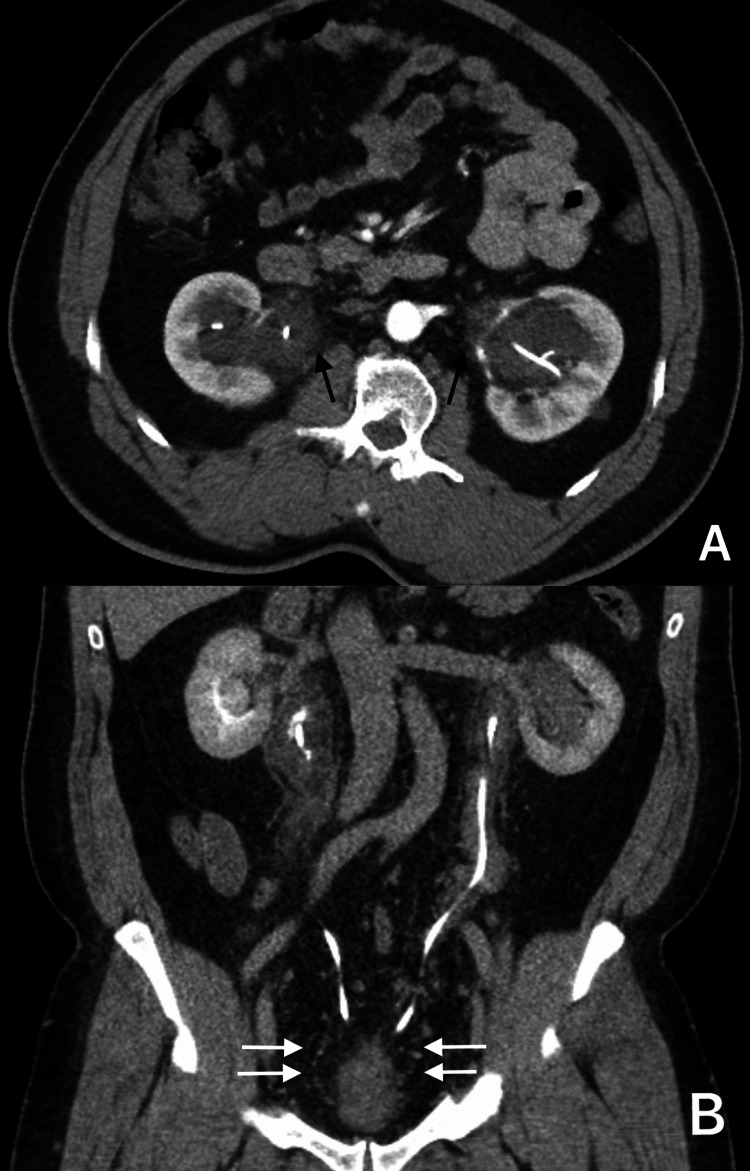
Pre-operative CT (axial and coronal view) (A) axial view, (B) coronal view. Pelvic lipomatosis (white arrows) causing moderate bilateral hydroureteronephrosis (black arrows). The finding of bladder wall thickening with peri-vesical and periureteric stranding.

The abdominal and pelvic CT scan showed a pear-shaped elongated urinary bladder secondary to extensive pelvic lipomatosis (Figure 1). The finding of bladder wall thickening with peri-vesical and periureteric stranding. Marked narrowing and compression of the rectum by the pelvic fat. Moderate bilateral hydroureteronephrosis with the bilateral double-J stent in place (Figure 2).

**Figure 3:**
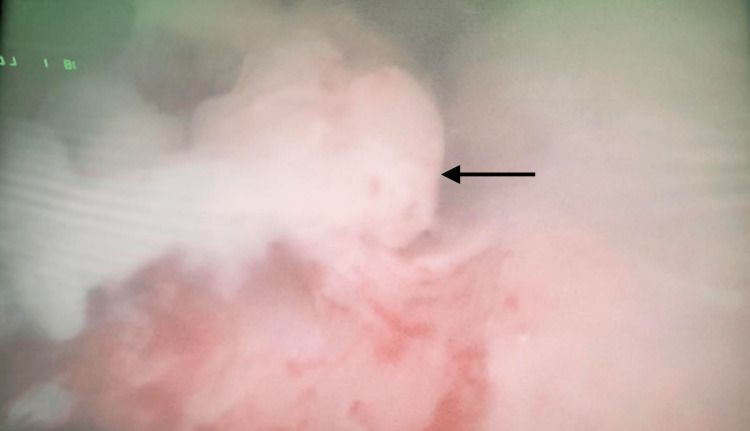
Cystoscopy image Cystoscopy showed several bullous lesions over the bladder, neck and trigone. Biopsy revealed cystitis glandularis (black arrow).

The patient underwent cystoscopy which showed an elongation of the prostatic urethra with a high bladder neck. There were several bullous lesions over the bladder neck and trigone without the involvement of the ureteric orifices. A tissue biopsy of the lesions demonstrated cystitis glandularis (Figure 3).

**Figure 4:**
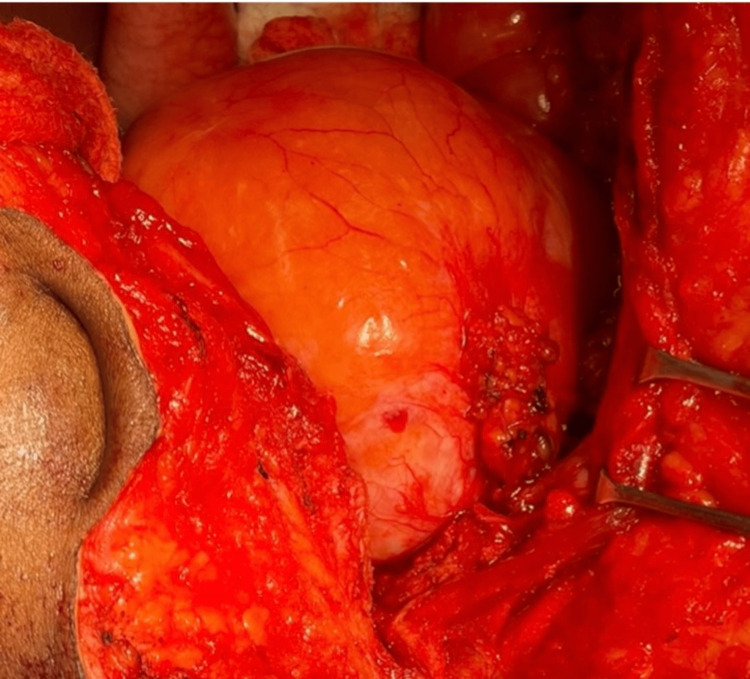
Intra-operative picture of the bladder

A laparotomy was performed through a midline incision, and the bladder was found high reaching intraperitoneal space (Figure 4). Bilateral ureters were identified. Release of adhesions with the removal of periureteric and perivesical fat. The proliferated fat around the ureter and bladder was extirpated and sent for histopathologic examination. Both ureters were dissected at the bladder junction, and the bladder incisions were closed using 3-0 absorbable sutures. The bilateral ureters were reimplanted at the bladder dome in an extravesical refluxing approach. No spatulation was needed as the lumens of the ureters were wide. The anastomosis was performed in a tension-free manner using a 4-0 absorbable suture over a double J stent. A pelvic drain and foley catheter were inserted. The operative time was 150 minutes and the estimated blood loss was 200 ml. Histopathology results confirmed the diagnosis of pelvic lipomatosis.

**Figure 5:**
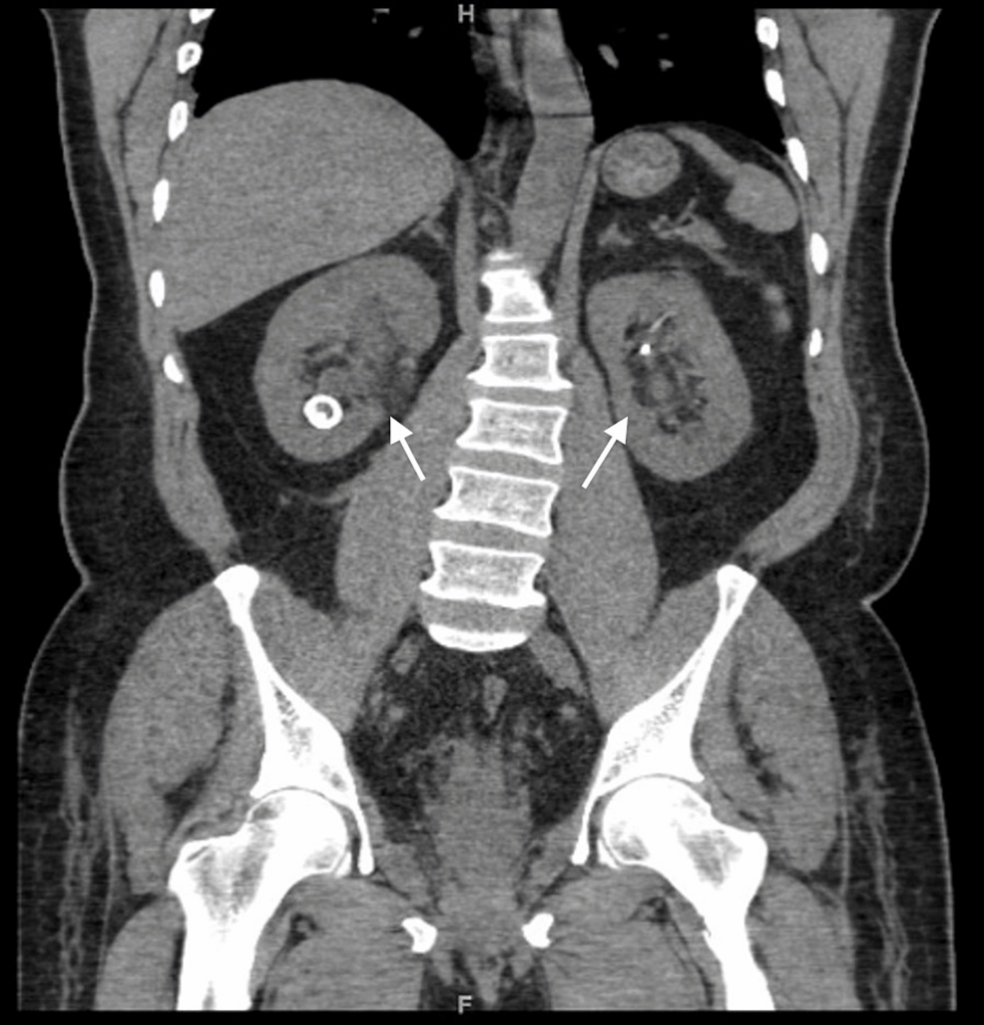
Post-operative CT image Marked improvement of bilateral hydronephrosis (white arrows) after ureteral reimplantation.

Postoperatively, the patient was ambulated and started on an oral diet on postoperative day 2. Repeated CT showed marked improvement in bilateral hydronephrosis (Figure 5).

Unfortunately, on post-operative day 10 patient was arrested after he was diagnosed with a massive pulmonary embolism and passed away despite being on a prophylactic dose of anticoagulant medications.

## Discussion

Pelvic lipomatosis is an uncommon disease associated with an overgrowth of fat in the pelvis and rectum areas [1]. Cystitis glandularis, cystitis cystica, or cystitis follicularis can be found in the majority of patients with the disease. These conditions are typically developed as a result of inadequate bladder drainage, forming a medium rich in protein fluid, and this is the ideal condition for proliferation. In particular, there are reports that cystitis cystica and glandularis could act as precursor lesions to adenocarcinoma [2].

Adequate monitoring and follow-ups are necessary for patients with cystitis cystica or glandularis so that bladder adenocarcinoma can be detected if present and transurethral resection can be repeated [2]. Clinical and radiological data are used to diagnose patients with this disease. Avoiding cystourethrogram, a type of contrast imaging, reveals whether the patient has a pear-shaped bladder, which is a symptom of the disease. Further, this imaging also reveals if the patient has an elevated bladder base or deviation of the ureters that can occur as a result of mass compression alongside related hydronephrosis. Computed tomography (CT) reveals the fat content surrounding the bladder and rectosigmoid for patients suffering from this disease. When patients have pelvic lipomatosis, this area is replaced by homogenous tissue of a low Hounsfield (−40 to −100 HU), and this often presents in a pear-shaped bladder [4].

There is limited research on the disease itself and its long-term effects, and so there are no set guidelines for patients to follow. This also means that there are numerous choices of treatment, such as watchful waiting or surgery that may involve a total cystectomy or urinary diversion [1]. Treatments with steroids, antibiotics, and radiation have proven to be unsuccessful [5].

According to Carpenter AA, patients who have pelvic lipomatosis can be categorized according to age, as the disease is typically indolent in patients who are older [1]. For older patients, therefore, management of the disease typically includes relief of urinary tract obstruction conservatively through the ureteral stent and transurethral resection of the prostate. However, the disease can be more severe in patients who are younger, and often presents in progressive ureteral obstruction that necessitates urinary diversion surgery. This surgery typically takes place as an ileal conduit or a ureterocutaneostomy [1]. Younger patients are often reluctant to undergo such procedures when facing this disease given the risks to their quality of life. Because of this, bladder-sparing surgery has been used with success in some cases as a treatment to improve the condition in the short term [5]. Halachmi et al. released a report on the first time a patient was treated with ureterolysis and ureteral reimplantation using an ultrasonic-assisted lipectomy device [6]. Further, Ali et al. released a report on a procedure where, through laparoscopy, pelvic fat was resected to preserve the bladder. With the removal of excess fat surrounding the pelvis, bladder pressure and capacity improves, meaning the patient may find their symptoms relieved to a certain extent [5].

## Conclusions

For patients suffering from pelvic lipomatosis and bilateral hydronephrosis, relief of obstructions to the urinary tract should be prioritized. This can be achieved through traditional surgery, such as a ureteral stent insertion, or more radical surgical options such as a total cystectomy and urinary diversion. There have been recent reports of success found in bladder-sparing techniques using mass extirpation and ureteral reimplantation.
